# Evaluation of the efficacy of sarolaner (Simparica®) in the prevention of babesiosis in dogs

**DOI:** 10.1186/s13071-017-2358-3

**Published:** 2017-09-06

**Authors:** Thomas Geurden, Robert Six, Csilla Becskei, Steven Maeder, Anne Lloyd, Sean Mahabir, Josephus Fourie, Julian Liebenberg

**Affiliations:** 1 Zoetis, Mercuriusstraat 20, B-1930 Zaventem, Belgium; 2 Zoetis, 333 Portage Street, Kalamazoo, MI 49007 USA; 3 Clinvet International, PO Box 11186, 9321, Uitsig Road, Universitas, Bloemfontein, 9338 South Africa

**Keywords:** Dogs, *Dermacentor reticulatus*, *Babesia canis*, Babesiosis, Transmission, Efficacy

## Abstract

**Background:**

Canine babesiosis is a clinically significant emerging vector-borne disease caused among others by the protozoan *Babesia canis*. The efficacy of sarolaner (Simparica®; Zoetis; at the minimum recommended label dose of 2.0 mg per kg bodyweight) in the prevention of babesiosis was evaluated in twenty-four dogs randomly allocated to either a placebo-treated group or one of two sarolaner-treated groups. At 21 or 28 days after treatment administration, dogs were infested with 50 ± 4 *Dermacentor reticulatus* ticks of which 25% were confirmed to be infected with *Babesia canis*. Blood samples were collected from each dog prior to tick infestation and weekly thereafter until 49 days after infestation. The blood was assayed for *B. canis* antibodies using an indirect immunofluorescence test (IFAT) and for *B. canis* DNA by PCR assay. A dog was a priori defined as *B. canis*-positive if it tested positive by both IFAT and PCR at any time during the study.

**Results:**

No treatment-related adverse reactions were recorded during the study. All placebo-treated animals displayed clinical signs due to babesiosis and tested positive on both IFAT and PCR. None of the sarolaner-treated animals displayed any clinical symptoms or tested positive on both IFAT and PCR, resulting in a 100% efficacy in the prevention of canine babesiosis (*P* = 0.0002).

**Conclusion:**

When given 21 or 28 days before tick infestation, a single treatment with sarolaner at the minimum recommended label dose of 2.0 mg per kg body weight prevented the transmission of *B. canis* by *D. reticulatus* to dogs.

## Background

Worldwide, dogs are exposed to a broad range of pathogens transmitted by ticks. One of the most significant and clinically relevant vector-borne diseases in dogs is known as piroplasmosis or babesiosis, caused mainly by *Babesia canis* [1]*.* The clinical signs associated with canine babesiosis vary from a mild transient illness to acute disease due to severe haemolysis that potentially leads to mortality. Clinical findings include anorexia, pale mucous membranes, icterus, pyrexia, and splenic enlargement. Next to these clinical signs, babesiosis in dogs is diagnosed by microscopy, IFAT or PCR or a combination of these techniques [2].

The protozoan parasite *B. canis* is transmitted mainly by adult *Dermacentor reticulatus* ticks, also known as the ornate dog tick. After *Ixodes ricinus*, *D. reticulatus* is the second most prevalent tick species in central Europe [3]. Although its prevalence varies depending on local environmental conditions, the distribution of *D. reticulatus* is expanding northwards in Europe [1, 4, 5]. For example, recent reports indicate that *D. reticulatus* has become widely established in Wales and South East England [6]. In addition to increased international travel of pets [1], climate change as well as changes in land use and host distribution, contribute to the expanding tick distribution. Furthermore, the total duration of tick questing activity over the year and the presence of *D. reticulatus* in winter months was shown to have increased in Belgium, Switzerland, Poland, Germany, Slovenia, Hungary and Slovakia [7–11].

In parallel with the geographical expansion of its vector, *B. canis* is increasingly reported in new endemic foci, for example in UK [12] and The Netherlands [13]. Furthermore, the prevalence of canine babesiosis is reported to be high in those regions where the tick is known to be common [14]. The expansion of *D. reticulatus* and the findings of significant *B. canis* infection rates justify the recommendation for year-round tick control measures in these areas [13]. Several treatments are known to kill *D. reticulatus* ticks and reduce *B. canis* transmission. In addition to the topically applied contact acaricides [15–17], systemic isoxazoline compounds have been shown to prevent *B. canis* transmission [18–20]. The objective of the current study was to evaluate the efficacy of a single treatment with sarolaner (Simparica®; Zoetis) at the minimum recommended label dose of 2.0 mg/kg in the prevention of *B. canis* transmission when dogs are challenged with infected *D. reticulatus* ticks 21 and 28 days after treatment.

## Methods

### Animals and ethical review

In total, 24 dogs (14 males and 10 females) were enrolled in the study. All dogs were either Beagles or mongrels, were between 1 to 8 years of age and weighed between 10.2 to 24.8 kg at enrolment. All dogs were found healthy upon a physical examination by the examining veterinarian and had been vaccinated and dewormed at most 14 days before treatment administration. All dogs had undergone a wash-out period sufficient to ensure that no residual ectoparasiticide efficacy remains from any previously administered compounds. Dogs were housed in individual pens except for the last 2 weeks (days 56 to 70 of the study) when dogs were housed in communal outdoor units. A suitable commercial dog food was given once daily to provide a maintenance diet. Fresh water was provided ad libitum. General health was observed twice daily.

### Treatment administration

Dogs were allocated to treatments and pens according to a randomized complete block design with one-way treatment structure. Blocking was based on pen location and the pretreatment body weight. Day 0 was defined as the day the dogs were dosed (Table 1). Dogs in group 1 were treated with placebo on days 0 and 7. Dogs in group 2 were treated with sarolaner on day 0 and with placebo on day 7, in order to evaluate efficacy 28 days after treatment. Dogs in group 3 were treated with placebo on day 0 and with sarolaner on day 7, in order to evaluate efficacy 21 days after treatment. Dogs were fed before dosing. The placebo or sarolaner tablet(s) were administered orally. Dogs were administered a single tablet or combination of tablets to achieve the minimum label dose of 2.0 mg sarolaner per kg body weight. Dogs were observed periodically for up to two hours for evidence of emesis and assessed for overall health prior to treatment on days 0 and 7, and 1, 3, 6 and 24 h after dosing.

**Table 1:** The study design with the different treatment groups, the number of animals in each group (*n*), the day of tick infestation and counts, and the treatment and sampling schedule

Treatment group	Treatment^a^		Day of tick infestation^b^	Days of tick counts^c^	Days of blood collections^d^
	Day 0	Day 7			
1 (*n* = 8)	Placebo	Placebo	28	29, 30 and 33	28 (prior to tick infestation), and 35, 42, 49, 56, 63, 70
2 (*n* = 7); treatment 28 days before tick infestation	Sarolaner (2 mg/kg)	Placebo			
3 (*n* = 8); treatment 21 days before tick infestation	Placebo	Sarolaner (2 mg/kg)			

^a^Dogs in group 1 received placebo tablets on Days 0 and 7. Dogs in group 2 received a sarolaner tablet on Day 0 and a placebo tablet on Day 7, to examine the efficacy 28 days after treatment. Dogs in group 3 received a placebo tablet on Day 0 and a sarolaner tablet on Day 7, to examine the efficacy 21 days after treatment

^b^Each dog was infested with 50 (±4) *Dermacentor reticulatus* ticks of which 25% were infected with *Babesia canis*

^c^Tick counts without removal were performed at 24 h (±2) and 48 h (±2) hours after tick infestation on Day 28. Tick counts with removal were performed on Day 33

^d^Blood collections for PCR analysis for detection of *B. canis* DNA and for IFAT for the antibody. On Day 28 only presence or absence of antibodies to *B. canis* was determined

Masking was accomplished by separation of functions of study personnel. All persons who made observations, conducted tick infestations and tick counts, performed laboratory analyses or performed general care for the dogs, were masked to the experimental treatments.

### Tick infestation and tick counts

On day 28 (21 days after sarolaner treatment in group 3 and 28 days after sarolaner treatment in group 2), each dog was infested with 50 (±4) viable, unfed adult *D. reticulatus* ticks at a 1:1 sex ratio. A laboratory-bred *D. reticulatus* tick strain, originating from Ireland and supplemented with ticks from the Netherlands in 2009 and 2012 was used for infestation. Ticks were infected with *B. canis* by feeding on a Clinvet colony dog with confirmed *B. canis* infection (PCR and IFAT). PCR testing [18] of a random sample confirmed that approximately 25% of ticks used for infestation were infected with *B. canis*. Ticks were applied between the shoulder blades. Dogs were restrained for ten minutes post application and then confined in an infestation chamber for approximately four hours to enhance tick attachment.

The tick count and categorization (free-attached, dead-alive, unfed-fed), without removal of the ticks, was conducted 24 (±2) and 48 (±2) hours after infestation by systematically examining the entire body. Each dog was examined for at least ten minutes. If ticks were encountered in the last minute, counting was continued in one-minute increments until no ticks were encountered. Tick counts and categorization were conducted on day 33 as described above, except that the ticks were removed.

### Clinical evaluation of babesiosis

Each dog received a physical examination prior to tick infestation on day 28 and again on days 35, 42, 49, 56, 63 and 70 for clinical signs of babesiosis, including but not limited to general body condition, respiration rate, heart rate, lethargy, anaemia, haematuria, anorexia, lymph node enlargement, splenic enlargement, ataxia and icterus. Additionally, rectal body temperature was measured daily for all dogs from day 34 to 70.

### Blood sampling and analysis

Blood samples were collected from each dog prior to tick infestation on day 28 for IFAT and again on days 35, 42, 49, 56, 63 and 70 for IFAT or PCR analysis. Blood samples were centrifuged, and serum was frozen until assayed for *B. canis* antibodies using a commercial test kit with *B. canis* antigen slides (MegaScreen® FLUOBABESIA canis Kit, Diagnostik Megacor, Hoerbranz, Austria). Total genomic DNA was isolated from whole blood samples taken after infestation using a commercial genomic DNA isolation kit (GeneJET Genomic DNA Purification Kit, Thermo Scientific, Waltham, MA, USA), with one modification: the samples were incubated for 16 h instead of the recommended one hour period during DNA extraction to maximize DNA release from the intracellular parasite(s) present in the host blood cells. PCR entailed the use of primers specific to the *B. canis* ITS region of the DNA. A PCR product of approximately 300 bp indicated the presence of the target region in the sample [18]. Positive, negative, no template as well as internal amplification controls were included in each PCR run.

If a dog had body temperature > 39.4 °C during the daily body temperature measurement, a clinical examination was conducted, and capillary blood was collected for blood smear examination from these dogs. Two blood smears were prepared from each dog. Blood smears were stained using a commercial Differential Quick Stain Kit (Modified Giemsa; Kyron Laboratories (Pty) Ltd., Johannesburg, South Africa) and microscopically examined for the presence of *B. canis*.

### Efficacy evaluation

The experimental unit for treatment was the animal. The efficacy of treatment in the prevention of *B. canis* transmission was evaluated based on the number of *B. canis*-positive dogs, defined as a dog testing serologically positive for *B. canis* antibodies and positive for *B. canis* by PCR analysis at any time-point during the 6 week follow-up period after infestation (day 35–70). The infection rate for each treatment group was calculated as the proportion of *B. canis*-positive dogs. A Fisher’s exact test was used to test for the overall treatment effect and to compare treatment groups to the control group. The percentage blocking efficacy was calculated for each treatment group separately.

The acaricidal efficacy was calculated based on mean live tick counts (free + attached) by treatment group and time point. Percent reductions relative to placebo based on the arithmetic and back transformed geometric least square means were calculated using Abbott’s formula:

$$ \% reduction=100\times \frac{mean count\ (placebo)\hbox{--} mean count\ (treated)}{mean count\ (placebo)} $$

Live tick counts from days 29 and 30 were analyzed using a general linear mixed model for repeated measures. The model included the fixed effect of treatment, time point and the treatment by time point interaction. The random effects included block, the interaction between block and treatment (animal term) and error. Live tick counts from day 33 were analyzed using a general linear mixed model. The model included the fixed effect of treatment and the random effects of block and error. Least squares means and standard errors were calculated, and 95% confidence intervals were constructed by treatment and time point. Geometric means (back-transformed means) were calculated from the least squares means. A priori contrasts were used to assess pair wise comparisons between treatments within time point. Treatment differences were assessed at each time point. All tests were done at the two-tailed 5% level of significance (*P* ≤ 0.05)*.*

## Results

All 24 animals enrolled in the study were dosed completely. No tablets were expelled, and no evidence of emesis was observed in any animal. Prior to infestation with the ticks on day 28, all animals were negative (PCR and IFAT) for *B. canis*. One dog in group 2 was excluded from the study, as the dog was mistakenly treated for babesiosis on day 35 due to an error in animal identification.

All placebo-treated dogs had fever within a week after tick infestation. Once infection with *B. canis* was confirmed by microscopic examination of a blood sample, the dogs were treated with diminazine aceturate IM (Berenil RTU®^;^ MSD Animal Health, The Netherlands) at 3.5 mg/kg body weight, dexamethasone (Kortico®) SC at 1 ml/10 kg body weight, followed by imidocarb diproprionate (Forray®65) IM at 1.2 ml/20 kg body weight the following day. All dogs recovered within 7 days of the onset of the clinical symptoms, but were found to be *B. canis*-positive during the follow-up period (Table 2): all were PCR-positive within seven days after infestation with *D. reticulatus*, seven of the eight placebo-treated dogs were positive for *B. canis* antibodies by 14 days after infestation (day 42), and the remaining dog seroconverted 28 days post infestation (day 56).

**Table 2:** The number of dogs positive for *Babesia canis* on blood smear, PCR and IFAT and number of dogs that were treated for babesiosis, as well as the number of dogs ever positive

Treatment group	Blood smear^a^	Rescue treatment	PCR^b^	IFAT	*B. canis* (Ever) positive^c^
1 (Placebo)	8 of 8	8 of 8	8 of 8	8 of 8	8 of 8
2 (Sarolaner 28 days earlier)	0 of 7	0 of 7	6 of 7	0 of 7	0 of 7^*^
3 (Sarolaner 21 days earlier)	0 of 8	0 of 8	3 of 8	0 of 8	0 of 8^*^

^a^Number of dogs positive for *Babesia canis* on a blood smear

^b^In the placebo group, 8 out of 8 dogs were PCR positive within 7 days after tick infestation. In the sarolaner-treated groups, 6 and 3 dogs had a faint amplification product 21 days after tick infestation. No amplification product was found in any of the dogs afterwards

^c^Includes all dogs that tested positive for *B. canis* by PCR and IFAT at any time during the study

^*^Number of positive dogs significantly lower than placebo (*P*-value = 0.0002)

None of the sarolaner-treated dogs displayed any clinical signs related to babesiosis or had any adverse reaction to treatment. Furthermore, none of the sarolaner-treated dogs were positive for *B. canis* antibodies at any time point during the study. In 9 sarolaner-treated animals (6 in group 2 and 3 in group 3) a faint amplification product was detected by PCR of the blood samples taken 21 days after tick infestation (day 49). The PCR was run in duplicate. Five of the nine dogs had a positive sample in both runs, and for 4 of these dogs, the sample failed to amplify in one of the two PCR runs. Several controls were included in the PCR, confirming that: (i) there was no PCR inhibition (internal control); (ii) the PCR mastermix was not contaminated with DNA (no template control); (iii) the primers in the mastermix performed adequately (positive control); (iv) canine DNA did not yield a false positive amplification product (negative control); (v) that the DNA extraction did not introduce DNA contamination (extraction control). A 100% efficacy (Fisher’s exact test: *P* = 0.0002) in the prevention of canine babesiosis was demonstrated, as no dogs tested positive for both IFAT and PCR during the study.The geometric (arithmetic) acaricidal efficacy was 93.0% (87.9%) and 94.6% (93.6%) on day 29 in group 2 and 3, respectively. On days 30 and 33, no live ticks were found on any of the treated dogs. Therefore, acaricidal efficacy was 100% in both treatment groups. The *D. reticulatus* tick counts were significantly lower (t-test: 6.88 ≤ *t*_(20)_ ≤ 72.73, *P* ≤ 0.0001) in both sarolaner-treated groups compared to the placebo-treated group on all counting days (Table 3).

**Table 3:** Live *Dermacentor reticulatus* tick counts: mean tick count, range, percent reduction and statistical comparisons (*P* value) at the different days (hours) after infestation

Days post-infection	Treatment group	Geometric (Arithmetic) mean	Range	Percent reduction^a^	*P*-value
1	1 (Placebo)	32.8 (33.1)	27–39	na	
	2 (Sarolaner 28 days earlier)	2.3 (4.0)	0–13	93.0 (87.9)	≤ 0.0001
	3 (Sarolaner 21 days earlier)	1.8 (2.1)	0–5	94.6 (93.6)	≤ 0.0001
2	1 (Placebo)	30.5 (30.9)	23–38	na	
	2 (Sarolaner 28 days earlier)	0	0–0	100	≤ 0.0001
	3 (Sarolaner 21 days earlier)	0	0–0	100	≤ 0.0001
5	1 (Placebo)	30.5 (30.9)	26–38	na	
	2 (Sarolaner 28 days earlier)	0	0–0	100	≤ 0.0001
	3 (Sarolaner 21 days earlier)	0	0–0	100	≤ 0.0001

*Abbreviation*: *na* not applicable

^a^Percent reduction calculated using the formula [(C–T)/C] × 100, where C is the geometric (arithmetic) mean of live tick counts for the control group and T is the geometric (arithmetic) mean of live tick counts for the treated group

## Discussion

The prevention of pathogen transmission through tick feeding has become an important characteristic for acaricidal products. Previously, sarolaner was shown to be efficacious in blocking the transmission of *Borrelia burgdorferi* and *Anaplasma phagocytophilum* by *Ixodes scapularis* [21]. In the current study, the ability of sarolaner to prevent the transmission of *B. canis*, causing one of the clinically most significant tick-borne diseases in dogs, was evaluated. Sarolaner is known to be highly effective against ticks, with a persistent efficacy of at least five weeks after a single treatment at the minimum recommended label dose [22]. Furthermore, a single treatment with sarolaner provides efficacy within 24 h for five weeks against *D. reticulatus* [23]. As such, sarolaner treatment has the potential to prevent *B. canis* transmission, as it takes 48 to 72 h after tick attachment for *B. canis* to be transmitted from an infected tick [24]. The rapid and consistent acaricidal efficacy of sarolaner against *D. reticulatus* was confirmed in the present study and indeed resulted in the prevention of *B. canis* transmission even at the end of the monthly dosing interval.

The experimental infection model used in this study was previously developed and described for the evaluation of the prevention of *B. canis* transmission [15–17, 19, 20, 25]. The advantage of an experimental model is that the challenge load can be standardized for all dogs [25], which is not the case under field conditions. A high number (*n* = 50) of ticks was used for infestation and ticks had a high (25%) infection rate with *B. canis*. In previous studies, infection rates of 8% to 16% were reported in experiments evaluating the efficacy of systemic products [18–20] and between 2% and 44% in studies evaluating the efficacy of topically applied acaricides [15–17, 25]. In the field, the *B. canis* prevalence in individual *D. reticulatus* ticks in Europe has been reported to be between 2.3% and 14.7% [26, 27]. The severe challenge, the evaluation of the efficacy towards the end of the treatment period and the treatment at the minimum recommended label dose in the current study, allowed evaluating a worst case scenario for *B. canis* transmission. In other studies with systemic acaricides, such as afoxolaner [18] and fluralaner [19, 20], the commercial band dosing was used.

In the current study, all placebo-treated animals developed clinical symptoms within one week after tick infestation. As *B. canis* infects the red blood cells and causes haemolysis, potentially resulting in severe disease and death, these dogs were immediately treated after they displayed clinical symptoms and tested positive by blood smear. The treatment did not impede the PCR diagnosis as the blood samples of all placebo-treated animals yielded an amplification product seven days after the infestation. Furthermore, all placebo-treated animals seroconverted. The infection pressure in this study was thus adequate to transmit *B. canis* infestation and cause clinical disease within a week after infestation.

As in a number of previous studies [15, 19, 20, 25] a dog was considered as *B. canis*-positive if both IFAT and PCR testing indicated infection. In the current study, a faint PCR amplification product was detected in 9 blood samples taken from sarolaner-treated dogs 21 days after infestation. The faint banding, as well as the inconsistent amplification in two consecutive PCR runs, suggests the presence of a low amount of template DNA in these samples. Although all PCR controls indicated that contamination during the DNA extraction or during PCR did not occur, DNA contamination of the blood sample prior to the start of the DNA extraction cannot unequivocally be excluded. Alternatively, the faint amplification products may result from the transmission of a low number of mature *B. canis* sporozoites present in the salivary glands of the ticks used for infestation [16]. Under normal life-cycle conditions, the tick’s attachment to the host’s skin initiates the maturation of the sporozoites in the salivary glands [15–17]. Under experimental conditions the maturation of sporozoites may be induced before the tick attachment [16], leading to a shorter transmission time than under field conditions [28]. The potential transmission of a low number of sporozoites did however not result in clinical disease, and none of these nine sarolaner-treated dogs seroconverted which would be expected in the event of successful transmission of viable protozoons, even at a low infective dose. Schetters et al. [29] indeed described that the onset of the immune response after *B. canis* infection but not the infection dynamics are dependent on the initial infectious dose. The extended follow-up for 42 days following infestation ensured that the sarolaner-treated animals with a faint PCR amplification product in the samples collected 21 days after infestations did indeed not seroconvert.

## Conclusion

The current study confirms the rapid and consistent acaricidal efficacy of sarolaner against *D. reticulatus* and its ability to prevent canine babesiosis caused by *B. canis,* under high challenge conditions and at the minimum recommended label dose. The persistent efficacy and rapid speed of kill for five weeks provided by sarolaner ensures a continued protection against tick infestation and subsequently against pathogen transmission in a monthly treatment regime.
